# The limited role of serum neurofilament light chain in predicting pain severity of patients with diabetic polyneuropathy

**DOI:** 10.1038/s41598-024-66444-9

**Published:** 2024-07-06

**Authors:** A-Sol Kim, Jong-Mok Lee

**Affiliations:** 1 Department of Family Medicine, School of Medicine, Kyungpook National University, Kyungpook National University Chilgok Hospital, Daegu, South Korea; 2 Department of Neurology, School of Medicine, Kyungpook National University, Kyungpook National University Hospital, 680 Gukchaebosang-ro, Jung-gu, Daegu, 41944 South Korea

**Keywords:** Diabetes mellitus, Axons, Peripheral nerve injuries, Diagnostic markers, Peripheral neuropathies

## Abstract

Pain is one of many complaints expressed by patients with diabetic polyneuropathy. However, no objective measure for pain severity has been available. Neurofilament light chains have been widely used for assessing axonal damage in the neuronal system. Hence, we sought to investigate whether neurofilament light chains can serve as a marker reflecting pain severity in diabetic polyneuropathy. We enrolled the patients with diabetic polyneuropathy. Serum concentrations of neurofilament light chain were then measured using a single-molecule array. Pain severity was evaluated using painDETECT and the Brief Pain Inventory. Moreover, laboratory results including, serum creatinine, HbA1c, and glomerular filtration rate. A correlation test was used to analyze each variable. A total of 42 patients were enrolled. Neurofilament light chain levels were unable to reflect current neuropathic pain severity. However, high levels of neurofilament light chain were a significant predictor of poor diabetes control (r = 0.41; *p* = 0.02) and kidney damage (r = 0.45; *p* = 0.01). Serum levels of neurofilament light chain could not reflect current pain severity but was strongly associated with kidney dysfunction and poor diabetes control. Other biomarkers that could predict pain severity need to be uncovered.

## Introduction

The International Association of the Study of Pain defines neuropathic pain as that caused by a lesion or diseases of the somatosensory system^1^. Several neurologic disorders, namely, central demyelinating diseases, radiculopathy, or peripheral neuropathy have been known to affect the somatosensory system. Therefore, neuropathic pain has become a major problem when treating residual symptoms of neurologic disorders. Furthermore, pain is defined as “an unpleasant sensory and emotional experience associated with, or resembling that associated with, actual or potential tissue damage”^2^; thus, pain itself can be influenced by personal experience. In line with this, several tools have been developed for assessing patient-reported pain^3–6^.

Diabetic polyneuropathy (DPN) is one of the most common neurologic disorders associated with neuropathic pain^7^. Approximately 50% of patients with DPN develop neuropathic pain, combined with paresthesia and sensory loss^7^, emphasizing the importance of assessing and managing neuropathic pain among affected patients. However, no objective measure exists for the severity of pain, although several questionnaires have been applied in the clinical practice.

Neurofilament light chains (NfLs) are cylindrical structures exclusively located in the neuronal cytoplasm that maintain the structural stability and radial growth of neurons^8^. Normally, neurons release minimal amounts of NfLs at the younger ages. However, once the axonal or neurons are damaged, the NfLs are abruptly released and pass freely between the cerebrospinal fluid (CSF) and the blood^8^. Thus, NfL has been widely used for the assessment of axonal damage in several neurological disorders^8–10^. NfL also plays a role in monitoring the disease course of multiple sclerosis, multiple systemic atrophy, and amyotrophic lateral sclerosis, as well as spinal muscular atrophy from clinical trials^9,11^.

The current study aimed to validate whether serum NfL can serve as an objective biomarker for pain severity in patients with DPN.

## Results

An overview of the participants’ characteristics and their correlation with NfLs is presented in Table 1, including etiology and risk factors. Most of the patients were diagnosed with confirmed diabetic sensorimotor polyneuropathy^12^. The painDETECT score was 14.14 ± 6.35, which indicated that most patients had less than moderate pain^13^. Among the seven items for painDETECT, tingling sensation was the most bothering symptom, followed by numbness. Concerning Brief Pain Inventory (BPI), the severity and interference scores were 4.05 ± 2.08 and 3.68 ± 2.73, respectively, which indicated mild pain^3^.

**Table 1:** Participant characteristics and their relation with serum neurofilament light chain.

Characteristics	Description Mean ± SD	Relation (NfL) *r*	N
Age	61.49 ± 11.10	0.1929	42
Sex, female (n, %)	10, 24%		42
Body weight	70.25 ± 13.78	− 0.1164	42
Male	70.21 ± 15.16		32
Female	70.40 ± 9.77		10
Type of diabetes (n)			
Type 1, type 2	0, 42		
Current smoker	31%		42
Current alcohol intake	38%		42
Weekly exercise (minutes, %)	415.28 ± 535.79, 43%	− 0.1523	18
Abnormality of neurophysiology (NCS, SSR, NCS and SSR, %)	14, 3, 16, 79%		33
NfL (pg/mL)	24.84 ± 12.63		42
Duration of painful neuropathy (months)	56.93 ± 72.85	− 0.07814	42
PainDETECT, seven items	14.14 ± 6.35	0.07814	42
Burning	2.17 ± 1.67	− 0.2215	42
Tingling	3.02 ± 1.68	− 0.03366	42
Pain by light tough	1.17 ± 1.43	0.02784	42
Electric shock-like pain	1.79 ± 1.70	0.2867	42
Pain on cold/heat stimulation	1.43 ± 1.33	0.04111	42
Numbness	2.52 ± 1.67	0.2407	42
Pain by slight pressure	2.05 ± 1.82	− 0.03479	42
BPI—severity	4.05 ± 2.08	0.04345	42
BPI—interference	3.68 ± 2.73	− 0.001165	42
Diabetes duration (years)	14.13 ± 10.3	0.2621	42
HbA1c	8.19 ± 2.01	0.4284*	35
LDL-C	83.58 ± 29.55	− 0.1517	36
C-peptide	2.31 ± 2.01	− 0.03799	26
GFR (mL/min/1.73 m^2^)	84.48 ± 30.66	− 0.3644*	34
Creatinine	1.05 ± 0.82	0.4042*	34
Fasting glucose	137.96 ± 41.53	0.2395	25

BPI, Brief pain inventory; GFR, glomerular filtration rate; LDL-C, low-density lipoprotein cholesterol; NCS, nerve conduction study; NfL, neurofilament light chain; SSR, sympathetic skin response (*p < 0.05, **p < 0.01).

### Association between serum NfL and patient-reported pain score

Serum NfL levels were not correlated with the painDETECT score (*r* = 0.07814; *p* = 0.6228), BPI severity (*r* = 0.04345; *p* = 0.7848), or BPI interference (*r* =  − 0.001165; *p* = 0.9942). Thereafter, we evaluated whether serum NfL levels were correlated with each item under painDETECT. However, no correlation was observed between serum NfL levels and each painDETECT item (Table 1).

### Association between serum NfL and HbA1c, glomerular filtration rate (GFR), and creatinine

**Figure 1:**
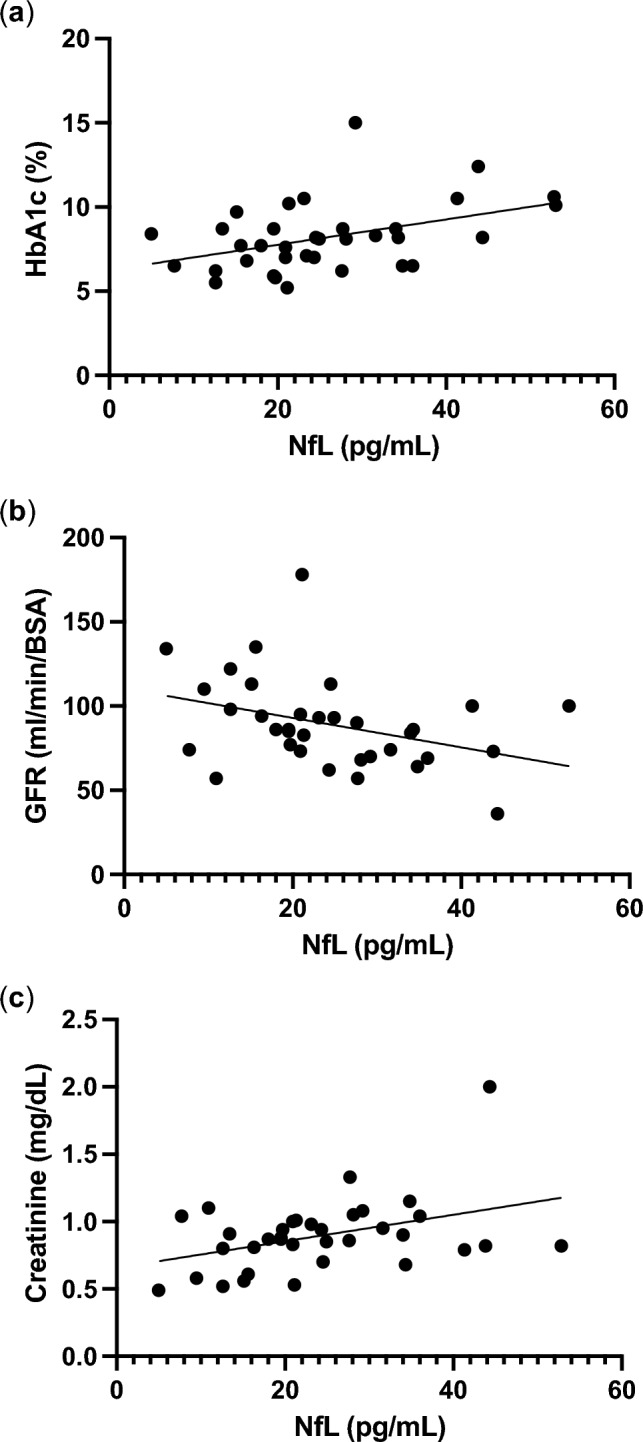
A positive correlation between serum NfL and HbA1c (**a**). Serum NfL was negatively correlated with glomerular filtration rate (**b**) and positively correlated with creatinine (**c**). NfL, neurofilament light chain.

HbA1c, GFR, and serum creatinine were found to be associated with serum NfL (Fig. 1). Increased NfL levels was positively correlated with increased HbA1c levels (*r* = 0.4284; *p* < 0.05). Moreover, serum NfL levels were negatively correlated with GFR (*r* =  − 0.3644; *p* < 0.05) and positively correlated with creatinine levels (*r* = 0.4042; *p* < 0.05). However, no correlation between serum NfL and other factors, such as disease duration, C-peptide, and fasting serum glucose level, were noted.

### Association between serum NfL and pain scores after adjusting for HbA1c, GFR, and creatinine

Multiple linear regression analysis was applied to investigate the correlation between serum NfL and pain scale while adjusting for effects of renal failure or current hyperglycemia (i.e., HbA1c, GFR, and creatinine). However, serum NfL was not correlated with patient-reported pain severity even after adjusting for the effects of hyperglycemia or renal failure (Table 2).

**Table 2:** The results of multilinear regression analysis of pain scale, HbA1c, GFR, creatinine to serum neurofilament light chain.

Variable	Unstandardized coefficients		Standardized coefficients	t	Sig	Collinearity statistics	
	B	Std. Error	Beta			Tolerance	VIF
(Constant)	0.366	20.537		0.018	0.986		
PD7	0.200	0.260	0.123	0.769	0.448	0.956	1.046
GFR	− 0.049	0.112	− 0.121	− 0.441	0.663	0.326	3.066
HbA1c	1.934	0.834	0.372	2.320	0.028	0.955	1.047
Cr	11.828	10.568	0.300	1.119	0.273	0.342	2.922
(Constant)	− 4.425	21.676		− 0.204	0.840		
BPI severity	0.774	0.928	0.139	0.834	0.411	0.881	1.135
GFR	− 0.030	0.110	− 0.073	− 0.271	0.789	0.335	2.985
HbA1c	1.953	0.833	0.375	2.344	0.027	0.952	1.050
Cr	14.701	10.834	0.372	1.357	0.186	0.324	3.082
(Constant)	0.538	20.625		0.026	0.979		
BPI interference	0.383	0.621	0.101	0.617	0.542	0.927	1.078
GFR	− 0.044	0.112	− 0.107	− 0.392	0.698	0.330	3.030
HbA1c	1.936	0.837	0.372	2.312	0.029	0.954	1.049
Cr	12.663	10.574	0.321	1.198	0.241	0.344	2.903

BPI, Brief pain inventory; Cr, creatinine; GFR, glomerular filtration rate; PD7, painDETECT 7 items

## Discussion

This cross-sectional study found no correlation between serum NfL levels and pain severity scores. However, our results showed that blood NfL levels were correlated with HbA1c, GFR, and serum creatinine, a finding consistent with that presented in previous reports^13^. NfL levels were not associated with pain scores, even after adjusting for factors such as HbA1c, GFR, and creatinine. Hence, we cannot definitively conclude whether serum NfL levels truly capture the subjective pain experienced by patients with DPN.

We selected painDETECT and BPI due to their comprehensive nature and extensive validation across diverse patient populations. The painDETECT is specifically designed to identify neuropathic pain components, making it a reliable tool for distinguishing neuropathic pain from other types of pain^14,15^. On the other hand, the BPI allows for a thorough assessment of pain severity and its impact on daily functioning, which is crucial for understanding the overall burden of pain in patients. In addition, both tools are easy to administer and interpret, enhancing their practical application in clinical settings^14,15^. Although more specific questionnaires, such as the NSP, MNSI, DN4, and McGill Pain Questionnaire, offer detailed insights into DPN symptoms, the validated effectiveness and simplicity of painDETECT and BPI make them highly valuable for comprehensive pain assessment in clinical practice^14–16^. Moreover, essential questions regarding symptoms are shared across questionnaires. Future studies could benefit from a combination of these tools to maximize diagnostic accuracy and patient care.

Concerning the pathomechanism of pain associated with DPN, the precise order of sensory nerve remains unknown^7^. However, evidence suggests that primary afferent hyperexcitability is a critical driver of pain following axon and Schwann cell damage or myelination^7^. Moreover, increased expression of voltage-gated sodium channel Nav 1.8 in sensory neurons has been associated with pain in DPN^7^. Furthermore, enhanced input from pain receptors increases synaptic transmission within the spinal cord, thereby amplifying nociceptive signaling to the same input^7^.

In our study, high serum NfL levels were correlated with increased HbA1c values. HbA1c, a form of hemoglobin linked to sugar, represents long-term glycemic status over the last 2–3 months^17^. Thus, our study suggests that long-term exposure to hyperglycemia induces axonal damage in the peripheral nerves. Similarly, patients with diabetes in the general population exhibit increased serum NfL levels^18^. In the pathomechanism of DPN, increased glucose levels promotes glucose metabolism via the polyol and hexosamine pathways, resulting in increased reactive oxygen species and inflammation^7^. Also, high serum glucose levels cause glycation of numerous proteins to produce advanced glycation end-products, triggering the release of proinflammatory molecules and free radicals^7^. As such, prevention of hyperglycemia will likely form part of the therapeutic strategies for patients with DPN.

Concerning the correlation between serum NfL and with renal function, our study demonstrated that serum NfL levels were negatively correlated with GFR and creatinine, a finding consistent with that reported in a previous study^13,19^. Although the precise mechanism by which kidney function affects blood NfL dynamics remains unknown, several hypotheses have been suggested. One hypothesis indicates that blood NfL is cleared by the kidneys^13^. Another possible mechanism is that the decline in renal function decreases the levels of erythropoietin and vitamin D, which exert neuroprotective effects^13,20^. Therefore, adjusting for renal function is required when assessing the associations between plasma NfL and other measures of neuronal damages^13,19^.

Our findings indicated that high serum NfL levels were positively correlated with poor diabetes control (elevated HbA1c levels) and kidney dysfunction (reduced GFR and increased creatinine levels), suggesting that NfL reflects overall metabolic and renal health rather than subjective pain severity. This finding is consistent with the understanding that hyperglycemia-induced oxidative stress and inflammation cause neuronal damage, which is reflected by increased NfL levels^7^. However, the subjective nature of pain, which is influenced by individual pain thresholds, psychological state, and other comorbidities, likely accounts for the lack of correlation between NfL and pain severity^1^. Moreover, the complex pathophysiology of DPN involves not only neuronal damage but also changes in ion channels, inflammatory processes, and microvascular complications, which collectively influence pain perception^7^. The positive correlation between NfL and markers of poor metabolic control and renal dysfunction underscores the role of NfL in the progression of DPN. These findings suggest that NfL cannot directly measure the subjective experience of pain in patients with DPN but can be a useful biomarker for assessing neuronal damage. Future research should explore additional biomarkers and more specific pain assessment tools to capture the multifactorial nature of pain in patients with DPN. Comprehensive management strategies that address both metabolic control and nerve health are essential for mitigating neuronal damage and improving patient outcomes.

The present study had several limitations. First, the enrolled sample size was small. The requirement to suspend drug administration for 2 weeks not only constrained the potential expansion of the sample size but also introduced the potential for recall bias. However, to obtain an accurate assessment of patient-reported pain severity in a drug-free state, suspension of drug administration was inevitable. Second, most patients reported mild to moderate pain. A sample of patients with a wider pain severity distribution might yield other results. Third, serum NfL levels of healthy individuals could not be elucidated. However, one previous study proposed normal cutoff values of serum NfL for healthy individuals^21^. Notably, the same study found that the upper limit of normal was higher among older individuals than among young healthy controls and that the mean serum NfL levels of enrolled subjects were higher than those of healthy controls by approximately 11 pg/mL when compared with the previous analysis^21^.

## Conclusion

Our findings showed that serum NfL levels were not correlated with subjective pain severity. This is most likely due to the complex pathways and mechanisms associated with pain and varying effects of personal experience. Therefore, investigation of only few components of the pain pathway in the patients with DPN cannot explain entire pain severity. Nonetheless, investigations into other biomarkers of neuropathic pain in patients with DPN may be worthwhile.

## Methods

### Patients

We prospectively recruited 42 patients with diabetic polyneuropathy at the department of neurology from September 2019 to May 2023. Diabetic polyneuropathy was diagnosed based on typical clinical symptoms or abnormal findings of nerve conduction study according to the criteria established by the Toronto Diabetic Neuropathy Expert Group^7,12^. Clinical symptoms entail the presence of characteristic symptoms, such as distal symmetric prickling, tingling, burning, aching pain, symmetric decrease of distal sensation, and absence of ankle reflexes^12^. Sympathetic skin response was investigated to identify small fiber neuropathy^22^. Patients with other conditions affecting peripheral neuropathies, namely, acute diseases, chronic alcoholism, concomitant cancer, radiculopathy, and nutritional deficiency, were excluded. Those who had a history of central nervous system disorders, such as ischemic stroke, were included that their condition remained stable 2 years after acute events.

All subjects voluntarily participated in this study after receiving sufficient explanation and signed an informed consent form approved by the ethics board of our institute (KNUH 2019-06-029). This study was conducted in accordance with the ethical principles for medical research involving human subjects stated in the Declaration of Helsinki.

### Study measures

#### Subjective pain severity

We evaluated subjective pain severity in the included patients using the Korean version of painDETECT and BPI-Short Form^3,4,23^. To eliminate the drug effects in pain management, all patients were drug naïve or did not receive any drugs for 14 days before completing the pain questionnaire.

The painDETECT comprises nine questions that evaluate the severity and pattern of pain and existence of neuropathic pain. Among the nine questions, one evaluates the temporal pain course pattern, whereas the others evaluate pain radiation^4^. Although all nine questions demonstrated predictive ability for neuropathic pain, principal component analysis identified seven sensory items establishing the data structure of the questionnaire. Therefore, the score for the seven-item painDETECT, which ranged from 0 to 35, was evaluated to determine pain severity^4,15^. The BPI comprises two main components, pain severity and pain-related interference of daily life. The severity component is scored from 0 (no pain) to 10 (most severe pain subjects can imagine), and subjects rated the severity of their pain via individual questions on the present, worst, least, and average pain, with the average of these four scores representing their pain severity index score. The pain-related interference score comprises the following seven domains: general activity, mood, walking ability, capacity for normal work, relationships with other people, sleep, and enjoyment of life. Respondents rated interference of daily life on a scale ranging from 0 (no interference) to 10 (complete interference), and the scores were added to obtain the total pain-related interference score^3^.

#### Serum NfL and other laboratory examinations

Blood samples were obtained from each patient to investigate serum NfL after completing the subjective pain severity questionnaire on the same day. In detail, 4 mL of blood was withdrawn from the patients, divided into 1.5-mL centrifuge tubes, and centrifuged at room temperature and 1300×*g* for 10 min to obtain the serum. Then, 500 μL of serum from each centrifuge tube was stored in a deep freezer at − 80 °C. The storage period did not exceed 6 months. The median level of serum NfL were measured twice through a single-molecule array (Quanterix Simoa NF-light Reagent Kit, MA, United States). Results of other blood tests, namely serum creatinine, HbA1c, low-density lipoprotein cholesterol, C-peptide, GFR, and fasting glucose were also included (Table 1).

### Statistical analysis

Correlations between continuous variables were calculated using the Pearson coefficient. Also, multiple linear regression was applied to adjust for other factors. A value of *p* < 0.05 indicated statistical significance. All analyses were conducted using Prism (GraphPad, Released 2022) and SPSS (IBM, version 26).

## Data Availability

The data used in this study are available from the corresponding author on reasonable request.
